# Evolution of a Large-Scale Community-Based Contraceptive Distribution Program in Kinshasa, DRC Based on Process Evaluation

**DOI:** 10.9745/GHSP-D-18-00205

**Published:** 2018-12-27

**Authors:** Julie H. Hernandez, Pierre Z. Akilimali, Mbadu Fidèle Muanda, Annie L. Glover, Jane T. Bertrand

**Affiliations:** a Global Health Management and Policy, Tulane University School of Public Health and Tropical Medicine, New Orleans, LA, USA.; b Kinshasa School of Public Health, University of Kinshasa, Kinshasa, Democratic Republic of the Congo.; c Programme National de Santé des Adolescents (PNSA), Kinshasa, Democratic Republic of the Congo.

## Abstract

In a context where distance, user fees, and health staff shortages constitute significant barriers to accessing facility-based family planning services, the use of community-based distributors (CBDs) as counseling and contraceptive providers has been tested in several resource-constrained environments to increase family planning uptake. In the capital city of the Democratic Republic of the Congo (DRC), Kinshasa, a massive CBD program (AcQual) has been implemented since 2014, with lackluster results measured in terms of the low volume of contraceptives provided. A process evaluation conducted in 2017 assessed the fidelity of implementation of the program compared with the original AcQual design and analyzed gaps in provider training and motivation, contraceptive supplies, and reporting and monitoring processes. Its objective was to identify both theory and implementation failures in order to propose midcourse corrections for the program. The mixed-method data collection focused on the CBDs as a pivotal component of the AcQual program with 700 active CBDs interviewed. In addition, 10 in-depth interviews were conducted with clinical personnel, local health program managers, and project partners to identify gaps in the AcQual implementation environment. Issues with CBDs' performance, knowledge retention, and commitment to program activities, as well as gaps in contraceptive supply chains and insufficient monitoring and supervision processes, were the main implementation failures identified. Inappropriate method mix offered by the CBDs (condoms, pills, and CycleBeads only) and chronic overburdening of health care staff at the local level compounded these issues and explained the low volume of contraceptives provided through AcQual. Midcourse corrections included a more structured schedule of activities, stronger integration of CBDs with clinical providers and health zone managers, expansion of the mix of contraceptives offered to include subcutaneous injectables and emergency contraceptive pills, and clarifying reporting and monitoring responsibilities among all partners. Findings from this process evaluation contribute to the limited knowledge base regarding “unwelcome results” by examining all the intervention components and their relationships to highlight areas of potential failures, both in design and implementation, for similar CBD programs.

## Abstract (precis)

Midterm process evaluation results indicated that design and implementation failures hindered the program's success, notably: (1) the short-acting methods provided by community-based distributors (CBDs) offered limited choice; (2) the nominal revenue retained from selling the methods provided limited motivation for the volunteer CBDs; and (3) the model was poorly coordinated with the existing clinical service system, partly because of challenging systems issues. In the revised model, the CBDs will also provide subcutaneous injectables and emergency contraceptive pills, retain more revenue from contraceptive sales, and have better interaction with the existing system including conducting monthly mini-campaigns to increase visibility and attract more clients.

Résumé en français à la fin de l'article.

## BACKGROUND

Family planning need in the Democratic Republic of the Congo (DRC) and its capital, Kinshasa, are among the highest in the world. Despite the progress achieved since the country's commitment to the Family Planning (FP2020) initiative in 2012, average fertility rates are high (6.6 per woman, according to the 2013–2014 Demographic and Health Survey) and modern contraceptive prevalence remains low (7.8%).^1^ In Kinshasa, the estimated unmet need for contraceptives among all women (i.e., the percentage of women who do not want another child for at least 2 years but are not currently using modern contraceptives) is 22.6%.^2^ This figure has been partially attributed to the lack of access to quality health facilities that can provide family planning services to women living in Kinshasa. Distance to facilities, frequent stock-outs, costs, and overburdened health care staff are indeed key obstacles to health care access in the DRC.^3^^,^^4^

Based on service delivery mechanisms designed and tested since the 1970s in numerous sub-Saharan African countries,^5^ including the DRC,^6^ to expand access to family planning services, the AcQual (“Access and Quality”) project is a large-scale community-based contraceptive distribution program designed and implemented to address some of these gaps. Its ultimate objective is to increase contraceptive uptake in Kinshasa (and later in Kongo Central). AcQual 1 was initially launched in 27 of the 35 health zones in Kinshasa in February 2014, then expanded in 2016 as AcQual 2 to cover 33 health zones (and an additional 12 health zones in Kongo Central, not covered in this study). The purpose of this article is to present findings of a process evaluation of the AcQual model as it was implemented between 2014 and 2017. It also describes how the community-based distribution model was redesigned to address the findings from the process evaluation.

## PROGRAM DESCRIPTION

### Context for Role and Deployment of CBDs in AcQual 1 and 2

Under AcQual, local health zone authorities recruited community-based distributors (CBDs) among volunteers who were often already involved in immunization or nutrition activities in their neighborhood. The only criteria for selection were neighborhood residence and completion of at least primary school. Two in-country implementing partners—the Association pour le Bien-Être Familial (ABEF), the International Planned Parenthood member affiliate in the DRC, and SANRU, a local faith-based organization involved in health care provision to marginalized Congolese communities since the 1980s—trained these individuals to provide family planning counseling and selected contraceptives (male condoms, oral contraceptive pills, and CycleBeads for use with the Standard Days Method) to previous and new family planning users living in their communities. Family planning clients who wanted methods not provided by the CBDs or who were experiencing side effects could be referred to nurses, also trained under the AcQual program, at nearby health facilities. The Health Zone Central Office (Bureau Central de la Zone de Santé, or BCZS), as the operational branch of Ministry of Health (MOH) programs in the DRC, and AcQual implementing partners shared the responsibilities for contraceptive resupply, reporting, and supervision of the distribution activities.

The intervention design centered on the CBDs, with the assumption that their roots and capacity to circulate in their communities would decrease the barriers associated with distance to facilities. It was thought that cost issues would be limited by the absence of registration or prescription fees typically charged by facilities in the DRC and the heavily discounted price of the contraceptives provided by the CBDs. The CBDs' familiarity with the community was seen as a stepping stone to recruit new modern contraceptive users who may otherwise have concerns about visiting a facility for family planning services. In addition, CBDs were encouraged to complete outreach activities (such as group discussions or canvassing), individual counseling, and referral to facilities, thus acting as a bridge to widen the pool of potential family planning users in their communities. However, because AcQual was designed to be integrated into the existing national health system of the DRC, to the extent possible, certain design choices had to be made to remain within the existing programmatic and policy boundaries. For example, in contrast to the situation in Ethiopia,^7^ it was agreed that the MOH in the DRC could not realistically offer a salary to the CBDs (considering that they pay regular public health facility employees sporadically). Similarly, given that the CBDs were not clinically trained, the range of methods they could provide was limited to pills, condoms, and CycleBeads, since in the DRC only doctors and nurses are typically permitted to provide injectables, implants, and intrauterine devices. Finally, reporting tools were designed to match those already used in the National Health Information System to avoid duplicating efforts, and it was decided that health zone staff and nurses at referral facilities would receive a portion of the contraceptive sales to increase buy-in chances.

The AcQual project design centered on community-based distributors providing condoms, pills, and CycleBeads in their communities at minimal cost to increase access to family planning.

### Initial Non-Systematic Monitoring

In the spring of 2016, the AcQual 2 team launched a non-systematic monitoring effort involving BCZS and facility visits, informal interviews with a convenience sample of about 60 CBDs, and a review of routine statistics. (This internal evaluation process was not part of a research activity submitted for approval to Tulane's Institutional Review Board but emerged organically during routine field visits to the project sites in Kinshasa. AcQual partners invited all CBDs and nurses operating in the health zone to be present during these visits and similar questions were asked during each of these sessions.)

The internal review raised some concerns about the seemingly low quantities of contraceptives (converted to couple-years of protection or CYPs) provided by the project's CBDs and facilities. Comparisons with the success of other community-based delivery strategies implemented between 2015 and 2016 in the DRC, such as use of medical and nursing school students to provide the subcutaneous injectable depot medroxyprogesterone acetate (DMPA-SC), under the brand name Sayana Press,^8^ further accentuated these concerns. The “focus group style” discussions with the CBDs about their experiences, motivations, and concerns with AcQual and the interviews with BCZS personnel supported a grounded theory approach to highlight several, non-mutually exclusive factors that could explain these lower-than-expected results for AcQual providers:

- The CBDs were not as active as initially expected.
- Frequent stock-outs may have resulted in reduced activity and missed opportunities for contraceptive provision.
- The CBDs may have distributed larger quantities of contraceptives that were underreported in AcQual monthly statistics.
- Some of the barriers to family planning use may not have been addressed adequately in the AcQual design.

An internal review 2 years into the project revealed low quantities of contraceptives provided by the community-based distributors.

### Design of the Formal Midterm Process Evaluation

To better understand the respective importance of these factors on the project's main objective to increase the modern contraceptive prevalence rate (mCPR) in Kinshasa and Kongo Central, the research team designed a midterm process evaluation. While midterm program evaluations often focus on measuring results obtained, without trying to analyze the actual implementation of the intervention intended to produce those results, the study described in this article specifically examined how the different components of the program were implemented.

Although there are few existing formal guidelines for process evaluations,^9^ the study framework typically proposes “to assess the quality, accuracy and fidelity to theoretical design and the relationships between the main program components.”^10^ This approach further enables program managers to clarify if unexpected or disappointing results are the product of “implementation” failure (the intervention design was appropriate, but the implementation was incorrectly or insufficiently conducted) or “theory” failure (the intervention was not adequately designed to address the issue).

The study examined the following components of the AcQual project design in terms of quality, accuracy, and fidelity:

- AcQual was designed to increase coverage of family planning services at the community level by deploying CBDs throughout Kinshasa.
- Once recruited, trained, and deployed, these CBDs had to be ready and able to provide quality family planning services to the populations living in their communities.
- The CBDs also needed to be continuously supplied with contraceptive methods to meet the needs and preferences of women living in their communities.
- CBDs and nurses at AcQual facilities had to be aware of the referral system and of their role in managing family planning clients counseled for clinical methods or management of side effects.
- Finally, ABEF and SANRU needed to properly monitor and supervise the CBDs to ensure that they received proper technical support, remained committed to AcQual activities, and reported routine service statistics in an accurate and timely manner.

The overall objective of the process evaluation was to identify gaps in the implementation of these components and/or unexpected responses to the intervention design from all actors involved, in order to formulate recommendations for midcourse corrections.

A process evaluation was conducted to identify any potential implementation failures or design failures.

## METHODS

The process evaluation used a mixed-methods design involving both quantitative and qualitative data, and findings were triangulated through routine reports and project documentation review. Key process indicators to assess the effectiveness and fidelity of AcQual implementation were defined in terms of:

- Levels of activities of the CBDs
- Volume of CYPs provided
- Frequencies of stock-outs
- Completeness and timeliness of routine statistics reports
- Frequency and quality of supervision
- Overall community and facility providers' satisfaction and engagement with the project

Because the CBDs were such a crucial component of the AcQual program, assessing their characteristics, performance, commitment, and satisfaction with their role in AcQual activities was central to the process evaluation. The research team thus proceeded to obtain a complete listing of all CBDs trained by ABEF and SANRU since the start of the project (N=870) and to systematically interview all who were still declaring themselves active with AcQual and who agreed to participate in the study. The CBD questionnaires were designed to collect multiple factors that could influence the CBDs' performance in contraceptive delivery, including socio-demographic characteristics such as gender, age, education, matrimonial status, number of children, and employment status (as proxies for burden on CBDs' time), knowledge and competence as family planning service providers, values and attitudes toward family planning provision, and commitment to the AcQual project.

In addition, the research team interviewed the 73 nurses trained under AcQual to handle referrals to fixed health care facilities, as well as the Chief Medical Officers and Community Activity Coordinator from the managing offices of the 33 health zones where AcQual CBDs were operational.

All quantitative surveys were administered electronically using the smartphone-based application OpenDataKit (ODK) and submitted to a protected server where the data were aggregated and extracted by the research team in the United States. This process allowed for an extremely quick turnaround in the evaluation results: the study was fielded in April 2017 and the results were presented at a dissemination meeting in July 2017.

Survey data from CBDs and nurses were triangulated with systematic review of the monthly reports available at the BCZS for the January–March 2017 period. Members of the research team visited each BCZS and documented the number of CBD monthly reports available compared with the number expected per AcQual reporting design (i.e., each CBD was expected to submit 2 reports every month, 1 report on services delivered and the other report on contraceptive stocks and flows).

Finally, the research team conducted 10 in-depth interviews with AcQual partners at the national level (MOH personnel and ABEF/SANRU representatives) and international level (UNFPA and DKT International, who were in charge of contraceptive supplies for the project).

Data collection for the process evaluation was approved by Tulane University Institutional Review Board (1029926-OTH) as well as by the Ethics Committee of the Kinshasa School of Public Health (#ESP/CE/014/2017).

## RESULTS

Of the 870 CBDs trained by AcQual partners since February 2014, 105 (12.1%) were no longer active (including 88 lost to follow-up and 7 deceased), 65 (7.5%) were still active but refused to be interviewed, and 700 (80.5%) declared they were still active and consented to be interviewed.

Overall, the number of days worked and CYPs distributed by active CBDs based on recall for the last month were low. CBDs declared “doing something related to the AcQual project” on average 8 days per month. When asked for the quantities of each contraceptive they remembered distributing over the past month, the mean quantity of CYPs provided amounted to 3.8, with a median of 1.3 (less than 1 set of CycleBeads per month). In addition, a quarter (25.3%) of the CBDs declared having distributed no contraceptives at all in the previous month.

The following findings break down the dimensions of AcQual 2 implementation that were believed to be crucial for CBDs to provide high quantities of contraceptives and quality family planning services at the community level, and they identify gaps in both model design and implementation.

### Adequate Coverage for Family Planning Service Provision

AcQual trained and deployed 3 CBDs per health area in 303 of the 381 (73.5%) health areas of the city. The project also trained an additional 73 nurses at referral facilities (2.2 per health zone on average). Thus, AcQual reached its stated objective to cover at least 70% of all health areas in Kinshasa. Based on population data provided by the Congo National Institute of Statistics, the average number of CBDs was 12.1 per 100,000 people at the health zone level (with a median of 9.3).

The project trained and deployed 3 community-based distributors per health area in nearly three-quarters of Kinshasa's health areas.

### Inadequate Readiness to Provide Quality Family Planning Services

All CBDs had been recruited and were operating in their own neighborhood and, following AcQual design, almost all (99.3%) had completed at least primary school, with 70.7% having completed high school or higher education levels. More than half (57.1%) were unemployed and one-third (33.8%) were already involved as community health workers for other programs. The average age of the AcQual CBDs was 45.9 years old, with 82.4% of them being over 35 years old. When asked about their main motivation to volunteer in AcQual, 42.1% of the CBDs expressed a desire to “help their community,” while 33.8% indicated that they were already involved in health outreach activities and 22.9% said they wanted to acquire new competences. Less than 1% initially mentioned money as a motivator.

The CBDs gave unanimously positive reviews to the training they received. However, results from the 15-question family planning knowledge test included in the questionnaire were mediocre, with 32% of the CBDs scoring below average and between a quarter and a third holding incorrect information for pregnancy screening, CycleBeads use, and medical eligibility for the pill. Compounding these poor knowledge levels, personal values (approximated by level of comfort in providing contraceptives to certain populations) may have hindered their level of activity: while 96.1% and 84.4% of the CBDs declared being “somewhat or very comfortable” with providing contraceptives to men and unmarried women, respectively, a quarter of them (23.4%) felt “somewhat or very uncomfortable” selling contraceptives to youth under 18 and almost half (47.4%) declared being “somewhat or very uncomfortable” providing family planning services to youth under 15. (Considering that this question has a strong desirability bias, the actual figures may possibly be higher.) Overall, 78.3% of the CBDs would have liked a refresher training, particularly on how to recruit new acceptors and how to refer women to facilities for management of side effects.

### Recurring Stock-Outs

Contraceptive stock-outs were one of the most prevalent issues reported by the CBDs. More than three-quarters (77.3%) had experienced at least 1 stock-out since the beginning of the project, with 75.4% stating that they were “sometimes or often” stocked out of condoms, and about half declaring being “sometimes or often” stocked out of pills (48.2%) and CycleBeads (46.2%) (Table 1). In addition, only 46.7% of the CBDs reported currently having at least one family planning counseling material (e.g., flyers, print job aids).

**TABLE 1.** Contraceptive Stock-Outs and Resupplies Reported by CBDs, Kinshasa, DRC, 2014–2017

	Condoms	COCs	POPs	CycleBeads
Stocked out often	41.4%	20.3%	22.7%	24.2%
Stocked out sometimes	34.0%	30.3%	23.1%	22.6%
Stocked out once	10.0%	13.1%	11.3%	13.7%
Never stocked out	14.6%	36.2%	42.9%	39.6%
Reported the stock-out	81.3%			
Resupplied	74.9%	70.4%	58.3%	57.2%

Abbreviations: CBDs, community-based distributors; COCs, combined oral contraceptives; DRC, Democratic Republic of the Congo; POPs, progestin-only pills.

Contraceptive stock-outs were one of the most prevalent issues reported by the community-based distributors.

The relatively rarer occurrence of stock-outs for pills and CycleBeads could be indicative of a lower demand for these types of contraceptives, a hypothesis compounded by CBDs' perception of women's preferences in their community. According to them, less than a quarter of all women would prefer to use CycleBeads (23.6%), the pill (21.7%), or condoms (18.9%) as their main contraceptive method. The perceptions of the CBDs are consistent with results from recent population surveys that show higher levels of use of implants, injectables, and emergency contraception.^2^ Qualitative data extracted from the CBD interviews indicated their frustration with their inability to offer these methods, which can be delivered in the DRC only by medically trained health care staff.^7^

### Weak Interaction With Clinical Services

The reported disconnect between the methods provided by the CBDs and the existing demand from potential family planning users could have been corrected within the AcQual project design, as long as the referral system to AcQual health facilities was operational and effective. The majority of CBDs (89.8%) stated that that they had referred “some” or “several” women to a health structure since the beginning of the project, with 71.1% indicating they referred women to the facility designated by AcQual partners during the training. In the majority of cases, women were referred because they wanted a method not provided by the CBD (Table 2). The second most frequently cited referral reason, mentioned by 29.9% of CBDs, was management of side effects.

**TABLE 2.** Assessment of the AcQual Referral System by CBDs and Facility-Based Nurses, Kinshasa, DRC, 2017

	CBDs	Nurses
Can identify counterpart in referral system	88.4%	54.7%
Frequency of interactions between CBDs and referral facilities		
Often	13.7%	31.4%
Sometimes	34.3%	31.4%
Rarely	42.6%	8.6%
Never	7.3%	27.1%
Reasons for referral to facilities		
Method not provided by CBD	83.9%	67.1%
Management of side effects	29.9%	40.0%
Preference for health facilities	11.3%	22.9%
Method provided by CBD but stocked out	9.1%	17.1%
Implants/IUD removal	—	21.4%
Other	7.4%	—
Does not know	3.3%	—
Perceived approval of CBD activities by health facility personnel		
Very positive	96.6%	70.0%
Somewhat positive		17.1%
Somewhat negative	2.1%	0.0%
Very negative		7.1%
Does not know	1.2%	5.7%

Abbreviations: CBD, community-based distributor; DRC, Democratic Republic of the Congo; IUD, intrauterine device.

In the majority of referral cases, community-based distributors reported that women were referred because women wanted a method they could not provide themselves.

Only half of the nurses (54.7%) could identify “all or most” of the CBDs operating in their health zone. But a higher percentage (62.8%) stated that they interacted between once a week and once a month with some CBDs in their health zone, which may indicate that nurses tend to always interact with the same individuals, while others remain under their radar.

Overall, the work of CBDs was perceived as mutually beneficial (by 96.6% of the CBDs and 87.1% of the nurses). Among the 7.1% of referral nurses holding “very negative” opinions of the work of CBDs, most were concerned with clinical errors made by CBDs in administering contraceptive methods and deceitful attitudes of CBDs who, as one nurse described, “present themselves as ‘doctors’ when they have not received adequate training.”

### Weak Support and Supervision of Community-Based Distributors

For AcQual to reach its stated objectives, the project needed to monitor and supervise CBD activities for the purpose of providing adequate technical support, ensuring CBD retention and motivation, limiting contraceptive stock-outs, and ensuring the completeness and timeliness of routine service statistics monitoring. The latter was another weak point of AcQual implementation with only 32.5% of the monthly reports being available at the BCZS for the January–March 2017 period. Only 48.4% of the CBDs were aware that there were 2 forms to be completed each month (one for family planning services and the other for contraceptive stocks, following the formats and requirements of the National Health Information System in the DRC), and 46.9% reported difficulties in completing and transmitting their reports to the BCZS each month. The main reasons mentioned for this situation were the absence of clients (21.2%) and the lack of time to complete (14.4%) or deliver (15.4%) the report to the BCZS. With 93.8% of the CBDs reporting in hard copy, distance and transportation costs, especially in the vast semi-rural health zones at Kinshasa's periphery, were significant barriers to routine statistics transmission. Conversely, 55.4% of the CBDs declared being out of blank forms to complete future reports. The BCZS staff interviewed during the evaluation pointed out that some of these statistics might be purposefully underreported since the CBDs are supposed to share the profits from contraceptive sales with both the BCZS and the AcQual partners. This situation might explain why 34.9% of the BCZS staff declared that the CBDs “rarely” or “never” reported their contraceptive sales.

In addition, while the CBD overall declared being “satisfied” or “very satisfied” (27.8% and 63.2%, respectively) of their relationship with the BCZS, the reported interaction between them seemed to vary significantly from one health zone to the next. The large majority (82.3%) of the CBDs declared attending at least one monitoring meeting at the BCZS, but just over half (56.9%) of BCZS staff did not know how many of these meetings were held over the past 6 months. In addition, 1 in 6 BCZS staff indicated that no monitoring meetings had been organized and only 4.6% declared that all 6 monthly meetings took place as planned under AcQual guidelines. At the next level of supervision (AcQual implementing partners, ABEF and SANRU), only a third (36.6%) of the CBDs recalled ever interacting with staff from these organizations after the training phase was completed. The numbers were higher for BCZS staff (83.3%), who would normally be visited by ABEF/SANRU at least once a month to provide service statistics reports and manage contraceptive resupply. However, when asked about the support they received from AcQual partners, 12.1% of the BCZS staff declared that it was “not very good” and 21.2% said that it was “not good at all.”

The overall perception of the project by its main implementing partners remained positive with 78.4% of the BCZS staff and 97.2% of the CBDs declaring being “satisfied” or “very satisfied” with their AcQual experience. However, qualitative comments at the end of both surveys listed recurring complaints about lack of adequate support and incentives. Multiple CBDs complained that:

*[We] are asked to perform a very difficult job almost pro bono. […] We don't have equipment, like rain boots and umbrella, we don't get much contraceptives and we don't have a salary or enough recognition to motivate us. […] Even just one of these things would improve our work. […] We also need new people to be involved. It's been three years for us and sometimes it feels like AcQual abandoned us a little.*

## DISCUSSION AND RESULTING CHANGES TO THE ACQUAL MODEL

A recent article published in the *Lancet*, “A call for transparency in the evaluation of global maternal health projects,”^11^ pointed out “the incongruity between successes—invariably reported at discrete program level—and the collective lack of progress in global maternal mortality” and suggested that underreporting of “unwelcome findings” in project evaluations might be partially responsible for this discrepancy. The findings presented in this article highlight both unwelcome “theory failures” and “implementation failures” that have hindered AcQual success in increasing access to quality family planning services at the community level.

The results specifically pointed toward 2 types of issues: (1) gaps specific to AcQual design or implementation that were not anticipated or controlled for when the project started, and (2) systemic issues related to the DRC health care delivery system that would require a broader, multipronged approach beyond the scope of AcQual to improve upon.

Considering the large body of existing literature on community health workers,^12^^–^^15^ some of the evaluation findings were not particularly surprising (e.g., lack of long-term commitment of volunteers, non-paid CBDs and their poor integration with health facilities). However, at the time it was initiated, AcQual was the first CBD program implemented in Kinshasa after a 20-year hiatus, and it was designed in close collaboration with the MOH, with the ultimate objective to become part of the national family planning service delivery system. Some of the project features were thus selected to avoid further burdening an already resource-constrained health system but were not always capable of compensating existing gaps.

Crucially, the initial assumption that the percentage of contraceptive sales going to the CBD would be a sufficient motivation to compensate their “volunteer” status and for them to be proactive in family planning service provision in their community was undermined by the purposefully low price of the contraceptives, along with the absence of incentives to report service statistics. In many cases, the benefits received from the monthly contraceptive sales could not cover the costs of public transportation for CBDs to attend monitoring meetings at the health zone office. Competing interests also compounded this situation since the CBDs are also often serving as health extension workers for other projects (e.g., immunization or HIV/AIDS prevention) that remunerate them directly.

In addition, existing medical norms in the DRC de facto limited the range of methods offered by AcQual CBDs, which did not match women's preferences for mid- to long-acting reversible methods such as injectables and implants. CBDs and other AcQual partners further perceived this as a missed opportunity since the higher retail price of these contraceptives could have supported a stronger commitment of the CBDs to community provision.

The short-acting methods that the community-based distributors could provide did not match women's preferences for mid- to long-acting reversible methods.

Finally, the routine provision model encouraging CBDs to provide contraceptives “whenever they had an occasion in the course of their daily lives in the community” proved haphazard in practice, in terms of unequal levels of CBD activities and gaps in the contraceptive resupply and reporting chains. In this regard, the process evaluation also provided evidence for all AcQual partners to acknowledge and discuss necessary organizational changes, including the identification and replacement of unproductive CBDs, the retraining of active CBDs (including value clarifications for family planning service provision to specific segments of the population such as youth and unmarried women), the formalization of reporting chains and supervision responsibilities from the BCZS to the AcQual managerial team at Tulane International, and the reallocation of budget items to increase coverage for monitoring and supervision activities.

### AcQual 3 Redesign

More crucially, findings from the process evaluation informed design and implementation changes that led to a revised CBD model (AcQual 3) to be implemented in Kinshasa and Kongo Central in 2019–2021. In particular, CBDs are now organized into a more rigorous family planning service provision schedule around monthly mini-campaign events known as *Samedi PF* (“Family Planning Saturday”), which brings AcQual 3 closer to the campaign days/advanced provision strategies implemented by national and international partners in the DRC as their main strategy for community-based contraceptive provision. The *Samedi PF* are expected to create a focal event that will help engage CBDs by gathering several community-based providers in one location (typically a market place) and thus potentially attract more clients. Moreover, these events will increase CBDs' visibility in the community by introducing them as local resources for family planning counseling and methods and provide them with an opportunity to report their service statistics, obtain contraceptives resupplies, and receive supervision without additional transportation costs.

In addition, the revenue from contraceptive sales is now going entirely to the CBDs to create a stronger financial incentive to participate in AcQual activities. This change should be complemented by the expansion of the method mix offered by CBDs, which in AcQual 3 will include DMPA-SC (Sayana Press) and emergency contraceptive pills, both in high demand and retailed at higher prices than other community-level methods. The addition of these 2 methods to the CBDs' method mix stems from separate Tulane-led research outlining the opportunities for community-based provision of these methods,^16^^,^^17^ and illustrate how pilot studies and operational research (such as this process evaluation) can be leveraged to improve program design and implementation.^18^

In the revised project model, the method mix offered by community-based distributors has been expanded to include subcutaneous injectables and emergency contraceptive pills.

However, some of the challenges identified in the midterm evaluation are partially beyond the scope of a single project such as AcQual. Chronic shortages and gaps in the contraceptive supply chain are a recurring issue in the DRC, particularly with the increase in demand for contraception over the past few years. Facility and community-based health workers tend to be overburdened by health care duties and multiple demands on their time for reporting project and program data.^19^ Despite the efforts engaged to closely integrate AcQual activities to public health programs implemented at the health zone level (e.g., relying on the BCZS for CBD recruitment, involving the MOH in their training, using standard forms for service statistics reporting), the project's activities, contraceptive resupply protocols, and reporting demands created redundancies and competing demands on health staff's already limited time and energy. Future efforts and modifications to the AcQual approach should address “increasing government ownership and leadership, limiting external inputs, and institutionalizing interventions within existing structures.”^20^

### Limitations

The existing literature on process evaluations converges on the absence of a systematic framework^9^^,^^10^ and the overwhelming and possibly redundant amount of information they tend to generate. This study focused on comparing the actual performance of the program to the model of how it was expected to function. In doing so, the research team measured fidelity to design and the providers' readiness to serve, perceptions, and level of satisfaction but did not capture other dimensions that process evaluation may capture such as client exposure and satisfaction.

Moreover, the lack of data on the performance of each individual CBD (measured by volume of contraceptive distributed) forced the research team to rely on ad hoc quantitative indicators created for this evaluation (number of days worked, volume of methods provided, and knowledge score) based on recall data provided by the CBDs themselves, which may be biased either toward overreporting (desirability bias) or underreporting (if they believed this would lead to more direct support or if they did not report sales to keep the benefits) their contraceptive distribution activities. The very gaps recorded in the reporting and supervision of family planning service provision may also be contributing to this perceived poor performance of the CBDs.

In addition, the focus on CYPs as a measure of AcQual successful implementation ignores dimensions of service quality (screening of family planning clients and adequate counseling) that are crucial not only to method adoption but also to long-term use.

Finally, this focus on CYPs may be overlooking other important aspects of the CBDs' work to promote access to contraceptives (e.g., outreach, individual counseling) and increase knowledge of, and demand for, family planning services in the community.

## CONCLUSION

The family planning supply environment in Kinshasa has been evolving rapidly since 2012.^21^^–^^23^ Multiple national and international partners now overlap, and sometimes compete for, contraceptive delivery using diverse pricing strategies, intervention scales, and clinical or community service points. As a result, the attribution of the slow but steady increase in mCPR in Kinshasa to a specific project or program is impossible, and AcQual is no exception. However, the preference given to a process evaluation for the project, and the presentation of its results to all family planning partners operating in Kinshasa in July 2017, offered an opportunity to isolate and assess intervention components that may be shared by other projects and programs (e.g., use of different cadres of community health workers, social marketing approach of contraceptive pricing) and to clarify their interaction and identify environmental factors and responses associated with variation in the project outcomes.

In the case of AcQual 2, in addition to systemic gaps in the DRC health care system, both theory failures (inappropriate method mix and inadequate incentive scheme to maintain CBDs' commitment) and implementation failures (contraceptive logistics gaps and insufficient monitoring and supervision of CBDs' activities) were responsible for the poor outcomes in terms of CYPs provided. Once these failures were identified, Tulane and its partners used the evaluation's findings to inform key design and implementation changes to the model, now AcQual 3, which is currently under implementation in the DRC (2019–2021).

While there are now growing concerns over scaling up and sustainability of community-based family planning provision models in resource-constrained environments, process evaluations such as the one presented in this article are an important contribution. They highlight how scientific evidence, donor strategies, and national or local situations inform initial design choices, which after field testing may prove inadequate. Innovation and lessons shared from other projects can help correct some of the identified gaps. However, the realistic integration of these models to existing national health systems (beyond the successful piloting of self-contained and resource-intensive projects), requires inventive compromises between strengthening the most effective components of the model and adapting to chronic failures of these systems.

## Supplementary Material

GHSP-D-18-00205-FR.pdf

## Notes

**En français**

**Évolution d'un programme à large échelle de distribution à base communautaire de contraceptifs à Kinshasa, RDC, sur la base d'une évaluation de processus**

Les résultats de l'évaluation de processus à mi-parcours ont indiqué que des lacunes dans la conception et la mise en oeuvre du programme ont limité sa réussite, en particulier : (1) les méthodes à courte durée d'action offertes par des distributeurs à base communautaire (DBCs) ne représentaient qu'un choix limit ; (2) le revenu symbolique tiré de la vente des méthodes n'étaient pas suffisant pour motiver les DBC volontaires ; et (3) le modèle était mal coordonné avec l'offre de services cliniques existante, partiellement à cause des challenges posés par le système sanitaire. Dans le modèle révisé, les DBC fourniront également des contraceptifs injectables sous-cutanés et des pilules contraceptives d'urgence, recevront un meilleur revenu de la vente des contraceptifs, et leurs activités seront mieux intégrées avec le système sanitaire, y compris par le bias de mini-campagnes mensuelles qui permettront d'accroître leur visibilité et d'attirer potentiellement plus de client(e)s.

**RÉSUMÉ**

Dans un contexte où l'éloignement, les frais cliniques, et les ressources humaines limitées constituent des barrières significatives à l'accès des services de planification familiale dans les structures de santé, l'utilisation de distributeurs à base communautaire (DBC) comme prestataires de conseils et de contraceptifs a été testée dans plusieurs environnements dont les ressources sont limitées, afin d'accroître l'adoption de la planification familiale. Dans la capitale de la République Démocratique du Congo (RDC), Kinshasa, un large programme de DBC (AcQual) a été mise en œuvre depuis 2014, avec des résultats médiocres en termes des volumes de contraceptifs fournis. Une évaluation de processus menée en 2017 a mesuré la fidélité de la mise en œuvre du programme par comparaison avec le design original d'AcQual et a analysé les lacunes concernant la formation et la motivation des prestataires, l'approvisionnement en contraceptifs, et les processus de supervision et de rapportage. L'objectif de l'évaluation était d'identifier les échecs de conception comme de mise en œuvre du programme afin de proposer des corrections à mi-parcours. La méthode mixte de collecte des données s'est concentrée sur les DBC, dans la mesure où ils sont une composante centrale du programme AcQual, et 700 DBC actifs ont été interviewés. De plus, 10 entretiens en profondeurs ont été menés avec les personnels cliniques, les gestionnaires des programmes de santé locaux, et les partenaires du projet afin d'identifier les lacunes organisationnelles pesant sur la mise en œuvre d'AcQual. Les principaux problèmes identifiés avaient à voir avec les performances des DBC, leur niveau de connaissance, et leur implication auprès des activités du programmes, ainsi qu'avec des lacunes dans les chaines d'approvisionnement en contraceptifs et des insuffisances dans les processus de supervision et de rapportage. La gamme contraceptive proposées par les DBC (limitée aux préservatifs, pilules et colliers du cycle) s'est révélée inadéquate et la surcharge de travail chronique pesant sur le personnel de santé au niveau local a aggravé ces problèmes tout en expliquant les faibles volumes de contraceptifs fournis par l'intermédiaire d'AcQual. Les corrections à mi-parcours incluent un calendrier plus structuré des activités, une meilleure intégration des DBC avec les prestataires cliniques et les responsables des zones de santé, une expansion de la gamme des contraceptifs offerts, qui inclue désormais les injectables sous-cutanés et les pilules contraceptives d'urgence, et une clarification des responsabilités de supervision et de rapportage parmi les partenaires. Les conclusions de cette évaluation de processus contribuent à la base limitée des connaissances sur les « résultats malvenus » en examinant l'ensemble des composantes de l'intervention et leurs relations pour mettre en évidence les zones d'échecs potentiels, en matière de conception comme de mise en œuvre, pour des programmes similaires de DBC.

## Notes

**Peer Reviewed**

**Funding**: David and Lucile Packard Foundation (Grant # 2017/65766).
