# Supplementary material for: Evolution of a Large-Scale Community-Based Contraceptive Distribution Program in Kinshasa, DRC Based on Process Evaluation
Source: Glob Health Sci Pract. 2018 Dec 27;6(4):657–67. doi: 10.9745/GHSP-D-18-00205 (PMC6370360; doi:10.9745/GHSP-D-18-00205)
Supplement: GHSP-D-18-00205-FR.pdf [file GHSP-D-18-00205-FR.pdf]

GLOBAL HEALTH: SCIENCE AND PRACTICE
Dedicated to what works in global health programs
OPEN ACCESS

ARTICLE ORIGINAL

Évolution d'un programme de distribution à base communautaire à grande échelle à Kinshasa, en RDC, selon son évaluation de processus

Julie H. Hernandez,[c] Pierre Z. Akilimali,[b] Mbadu Fidèle Muanda,[c] Annie L. Glover,[a] Jane T. Bertrand[a]

Les résultats de l'évaluation de processus à mi-parcours indiquent que des échecs de conception comme de mise en œuvre ont limité le succès du programme, notamment: (1) les méthodes à courte durée d'action fournies par les distributeurs à base communautaire (DBC) n'ont offert qu'un choix limité; (2) les revenus nominaux perçus grâce aux ventes des méthodes contraceptives ont apporté peu de motivation aux DBC bénévoles; et (3) le modèle a souffert d'une faible coordination avec le système de services cliniques existant, en partie en raison de défis systémiques. Sous le modèle repensé, les DBC fourniront désormais aussi les injectables sous-cutanés et la contraception d'urgence, garderont une plus grande partie des recettes de leurs ventes de contraceptifs, et bénéficieront d'une meilleure interaction avec le système existant, y compris en menant des mini-campagnes mensuelles pour augmenter leur visibilité et attirer plus de clients.

RÉSUMÉ

Dans un contexte où l'éloignement, les frais cliniques, et les ressources humaines limitées constituent des barrières significatives à l'accès des services de planification familiale dans les structures de santé, l'utilisation de distributeurs à base communautaire (DBC) comme prestataires de conseils et de contraceptifs a été testée dans plusieurs environnements dont les ressources sont limitées, afin d'accroître l'adoption de la planification familiale. Dans la capitale de la République Démocratique du Congo (RDC), Kinshasa, un large programme de DBC (AcQual) a été mise en œuvre depuis 2014, avec des résultats médiocres en termes des volumes de contraceptifs fournis. Une évaluation de processus menée en 2017 a mesuré la fidélité de la mise en œuvre du programme par comparaison avec le design original d'AcQual et a analysé les lacunes concernant la formation et la motivation des prestataires, l'approvisionnement en contraceptifs, et les processus de supervision et de rapportage. L'objectif de l'évaluation était d'identifier les échecs de conception comme de mise en œuvre du programme afin de proposer des corrections à mi-parcours. La méthode mixte de collecte des données s'est concentrée sur les DBC, dans la mesure où ils sont une composante centrale du programme AcQual, et 700 DBC actifs ont été interviewés. De plus, 10 entretiens en profondeurs ont été menés avec les personnels cliniques, les gestionnaires des programmes de santé locaux, et les partenaires du projet afin d'identifier les lacunes organisationnelles pesant sur la mise en œuvre d'AcQual. Les principaux problèmes identifiés avaient à voir avec les performances des DBC, leur niveau de connaissance, et leur implication auprès des activités du programmes, ainsi qu'avec des lacunes dans les chaines d'approvisionnement en contraceptifs et des insuffisances dans les processus de supervision et de rapportage. La gamme contraceptive proposées par les DBC (limitée aux préservatifs, pilules et colliers du cycle) s'est révélée inadéquate et la surcharge de travail chronique pesant sur le personnel de santé au niveau local a aggravé ces problèmes tout en expliquant les faibles volumes de contraceptifs fournis par l'intermédiaire d'AcQual. Les corrections à mi-parcours incluent un calendrier plus structuré des activités, une meilleure intégration des DBC avec les prestataires cliniques et les responsables des zones de santé, une expansion de la gamme des contraceptifs offerts, qui inclue désormais les injectables sous-cutanés et les pilules contraceptives d'urgence, et une clarification des responsabilités de supervision et de rapportage parmi les partenaires. Les conclusions de cette évaluation de processus contribuent à la base limitée des connaissances sur les « résultats malvenus » en examinant l'ensemble des composantes de l'intervention et leurs relations pour mettre en évidence les zones d'échecs potentiels, en matière de conception comme de mise en œuvre, pour des programmes similaires de DBC.

[a] Global Health Management and Policy, Tulane University School of Public Health and Tropical Medicine, New Orleans, LA, USA.
[b] École de Santé Publique de Kinshasa, Université de Kinshasa, Kinshasa, République Démocratique du Congo.
[c] Programme National de Santé des Adolescents (PNSA), Kinshasa, République Démocratique du Congo.
Correspondance à Julie Hernandez (hernanjulie@gmail.com).

CONTEXTE

Les besoins en planification familiale en République Démographique du Congo et dans sa capitale, Kinshasa, sont parmi les plus élevés au monde. En dépit du progrès accompli depuis l'engagement du pays au Sommet de l'initiative Family Planning 2020 (FP2020) en 2012, l'indice synthétique de fécondité du pays est élevé (6,6 par femme, selon l'Enquête Démographique et de Santé de 2013–2014) et la prévalence contraceptive moderne demeure faible (7,8%).[1] À Kinshasa, on estime à 22,6% les besoins non satisfaits en contraceptifs chez toutes les femmes (soit le pourcentage de femmes qui ne souhaitent pas avoir d'enfant dans les deux prochaines années *a minima*, mais n'utilisent actuellement aucun moyen de contraception).[2] Ce chiffre a été en partie attribué au manque d'accès aux structures de santé fournissant des services de planification familiale de qualité aux femmes vivant à Kinshasa. L'éloignement des structures de santé, les ruptures de stock fréquentes, les frais cliniques, et les ressources humaines limitées dans les structures de santé sont autant d'obstacles à l'accès aux soins en RDC.[3,4]

Fondé sur les mécanismes de prestation de services conçus et testés depuis les années 1970 pour élargir l'accès à la planification familiale dans de nombreux pays de l'Afrique subsaharienne,[5] dont la RDC,[6] le projet AcQual (« Accès et Qualité ») est un programme de distribution à base communautaire à grande échelle conçu et mis en œuvre pour combler certains vides dans l'accès aux soins. Son but principal est l'augmentation de la prévalence contraceptive moderne à Kinshasa (puis dans la province du Kongo Central). AcQual 1 a été initialement lancé dans 27 des 35 zones de santé de Kinshasa en février 2014, puis s'est élargi en 2016 sous le nom AcQual 2 pour couvrir 33 zones de santé (et 12 zones de santé additionnelles au Kongo Central, non incluses dans cette étude). Cet article présente les résultats d'une évaluation de processus du modèle AcQual mis en œuvre entre 2014 et 2017. Il décrit également comment ce modèle de distribution à base communautaire a été repensé en réponse aux résultats de cette évaluation de processus et pour en intégrer les changements subséquents.

DESCRIPTION DU PROGRAMME

Contexte du rôle et du déploiement des DBC sous AcQual 1 et 2

Sous AcQual, les autorités des zones de santé ont recruté des distributeurs à base communautaire (DBC) parmi des volontaires qui pour beaucoup étaient déjà impliqués dans les activités d'immunisation ou de nutrition dans leurs quartiers. Le seul critère de sélection était de résider dans un quartier couvert par l'initiative et d'avoir terminé au moins l'école primaire. Deux partenaires de mise en œuvre dans le pays -l'Association pour le Bien-Être familial (ABEF), membre affilié de la Fédération internationale pour la planification familiale (IPPF) en RDC, et SANRU, une organisation confessionnelle locale impliquée dans la prestation de soins de santé aux communautés congolaises marginalisées depuis les années 1980- ont formé ces personnes au conseil en planification familiale et à l'administration d'une sélection de produits contraceptifs (le préservatif masculin, la pilule, et le collier du cycle utilisé avec la Méthode des Jours Fixes) aux anciennes et nouvelles utilisatrices de la planification familiale vivant dans leurs communautés. Les clientes de la planification familiale désirant une méthode non fournie par les DBC, ou souffrant d'effets secondaires, pouvaient être référées à des infirmiers, également formés par le programme AcQual, dans des structures de santé à proximité. Le Bureau Central de la Zone de Santé (BCZS), branche opérationnelle des programmes du Ministère de la Santé de la RDC, et les partenaires de mise en œuvre d'AcQual se partageaient la responsabilité du réapprovisionnement des stocks de contraceptifs, du rapportage des données, et de la supervision des activités de distribution.

Le modèle d'intervention centré sur les DBC est fondé sur le postulat que leur enracinement dans la communauté, et leur capacité à y circuler, permettrait de réduire les barrières associées à l'éloignement des structures de santé. L'hypothèse était que les difficultés liées au coÃt seraient limitées par l'absence de frais d'inscription ou de prescription typiquement facturés par les structures de santé en RDC, et grâce au prix hautement subventionné des contraceptifs fournis par les DBC. Le fait que les DBC soient déjà connus de leurs communautés était perçu comme un tremplin pour recruter de nouvelles utilisatrices de la contraception moderne qui pourraient autrement être réticentes à l'idée de se rendre dans une structure de santé pour obtenir des services de planification familiale. Les DBC ont été encouragés à mener des activités de sensibilisation complémentaires (groupes de discussion ou porte-à-porte), du conseil individualisé, et à référer les clientes vers des structures de santé au besoin, servant ainsi de pont pour accroître la masse d'utilisatrices potentielles de la planification familiale dans leurs

communautés. Cependant, AcQual ayant été conçu pour être autant que possible intégré au système de santé national existant en RDC, certains choix relatifs à la conception même du programme ont dÃ être faits dans les limites des politiques et programmes existants. Par exemple, contrairement au cas de l'Éthiopie,[7] il avait été convenu que le Ministère de la Santé ne pourrait de manière réaliste offrir de salaire aux DBC (étant donné qu'il ne paie les employés titulaires des structures de santé publiques que de manière sporadique). De la même manière, les DBC n'ayant pas de formation médicale, la gamme de méthodes qui leur était permis de distribuer était limitée à la pilule, le préservatif et le collier du cycle, puisqu'en RDC, seuls les médecins et les infirmiers sont autorisés à fournir les injectables, les implants et les dispositifs intra-utérins. Enfin, les instruments de rapport de données avaient été conçus pour coïncider avec ceux déjà utilisés dans le cadre du Système d'Information Sanitaire National afin d'éviter la duplication d'efforts, et il avait été décidé que le personnel de la zone de santé et les infirmiers des structures de santé de référence percevraient une part des recettes de la vente des contraceptifs fournis par les DBC pour augmenter les chances de bénéficier de leur soutien.

Première évaluation (non systématique)

Au printemps 2016, l'équipe d'AcQual 2 a entrepris un effort de suivi non-systématique en impliquant les BCZS et incluant des visites de supervision dans les structures de santé, des entretiens informels avec un échantillon de commodité d'environ 60 DBC, et un examen des statistiques de routine. (Cette évaluation interne ne faisait pas partie de l'activité de recherche soumise à l'approbation du Comité d'éthique biomédicale de Tulane, mais a émergé de manière organique pendant les visites de terrain de routine sur les sites du projet à Kinshasa. Les partenaires d'AcQual ont invité tous les DBC et infirmiers opérant dans la zone de santé à être présents lors de ces visites, et des questions similaires ont été posées lors de chacune de ces sessions).

L'évaluation interne a soulevé plusieurs questions concernant les quantités apparemment faibles des contraceptifs (converties en années protection couple, ou APC) fournies par les DBC et les structures de santé participant au projet. Cette faible performance était d'autant plus préoccupante, en comparaison au succès d'autres stratégies de distribution à base communautaire mises en œuvre entre 2015 et 2016 en RDC telles que l'utilisation d'étudiants en médecine et en soins infirmiers pour fournir l'injectable sous-cutané depot medroxyprogesterone acetate (DMPA-SC), sous le nom de marque Sayana Press.[8] Les discussions de groupe menées avec les DBC sur leurs expériences, motivations et préoccupations concernant AcQual, et les entretiens conduits avec le personnel des BCZS, ont soutenu une approche de la théorie ancrée soulignant plusieurs facteurs (non incompatibles) qui pourraient expliquer des résultats en deçà du standard attendu pour les prestataires d'AcQual:

1. Les DBC n'étaient pas aussi actifs qu'anticipé.
2. Les ruptures de stock fréquentes pourraient avoir donné lieu à une réduction de l'activité et à des occasions manquées de distribuer des contraceptifs.
3. Les DBC pourraient avoir distribué de plus grandes quantités de contraceptifs, mais sous-déclarées dans les statistiques mensuelles d'AcQual.
4. Certaines barrières à l'utilisation de la planification familiale pourraient ne pas avoir été adéquatement prises en compte dans la conception du modèle AcQual.

Conception de l'évaluation formelle de processus à mi-parcours

Pour mieux comprendre l'incidence de chaque facteur sur l'objectif principal du projet d'augmenter le taux de prévalence contraceptive moderne (TPCm) à Kinshasa et dans la province du Kongo Central, l'équipe de recherche a conçu une évaluation de processus à mi-parcours. Tandis que les évaluations de programmes à mi-parcours se focalisent souvent sur la mesure des résultats obtenus, sans tenter d'analyser la mise en œuvre à proprement parler de l'intervention qui visait à produire ces résultats, l'étude décrite ici examine spécifiquement la manière dont les différentes composantes du programme ont été mises en œuvre.

Bien qu'il existe peu de recommandations formelles pour les évaluations de processus,[9] le cadre d'analyse de ce type d'étude propose généralement d' « évaluer la qualité, l'exactitude et la fidélité au concept théorique, et les liens entre les composantes principales du programme ».[10] Cette approche permet aux gestionnaires de programmes de déterminer la mesure dans laquelle des résultats inattendus ou décevants sont le produit d'échecs de « mise en œuvre » (la conception initiale de l'intervention était appropriée, mais la

mise en œuvre a été inadaptée ou insuffisante) ou d'échecs de « conception » (la conception initiale de l'intervention n'était pas adéquate pour résoudre le problème).

Cette étude examine les éléments suivants du modèle AcQual en termes de qualité, exactitude et fidélité:

- AcQual a été conçu pour augmenter la couverture des services de planification familiale au niveau communautaire en déployant des DBC dans toute la ville de Kinshasa.
- Une fois recrutés, formés et déployés, ces DBC devaient être préparés et capables de fournir des services de planification familiale aux populations vivant dans leurs communautés.
- Les DBC avaient également besoin d'être continuellement réapprovisionnés en contraceptifs pour répondre aux besoins et préférences des femmes vivant dans leurs communautés.
- Les DBC et infirmiers dans les structures de santé d'AcQual devaient connaître le système de référence et être conscients de leur rôle dans la gestion des clientes conseillées pour des méthodes cliniques ou dans la gestion des effets secondaires.
- Enfin, ABEF et SANRU devaient suivre et superviser adéquatement les DBC pour s'assurer qu'ils reçoivent un soutien technique adéquat, demeurent engagés à mener les activités d'AcQual, et rapportent leurs statistiques de services de routine de manière exacte et en temps opportun.

L'objectif général de cette évaluation de processus était d'identifier les lacunes de la mise en œuvre de ces composantes et/ou les réponses inattendues au modèle d'intervention initial de la part de toutes les parties prenantes concernées, dans le but de proposer des corrections à mi-parcours.

MÉTHODES

L'évaluation de processus a employé une méthode mixte de collecte de données à la fois quantitatives et qualitatives, et les résultats ont été triangulés via les rapports de routines et une analyse des documents du projet. Les indicateurs de processus clés pour évaluer l'efficacité et la fidélité de la mise en œuvre d'AcQual ont été définis comme suit:

- Niveau d'activités des DBC
- Volume d'APC fournies
- Fréquence des ruptures de stock
- Intégrité et ponctualité des rapports statistiques de routine
- Fréquence et qualité de la supervision
- Satisfaction générale et implication de la communauté et du personnel des structures de santé dans le projet.

Dans la mesure où les DBC constituaient un élément central du programme AcQual, cette évaluation de processus s'est essentiellement concentrée sur leurs caractéristiques, performance, engagement et satisfaction concernant leur rôle dans les activités d'AcQual. L'équipe de recherche a donc obtenu une liste complète de tous les DBC formés par ABEF et SANRU depuis le début du projet (N=870) et interviewé toutes celles et ceux se déclarant comme travaillant toujours pour AcQual et ayant consenti à participer à cette étude. Les questionnaires des DBC ont été élaborés de manière à collecter des données sur plusieurs facteurs qui pourraient influencer leur performance dans la distribution des contraceptifs, dont des caractéristiques sociodémographiques telles que le sexe, l'âge, le niveau d'études, le statut conjugal, le nombre d'enfants et la situation d'emploi (en tant que facteurs pesant sur le temps disponible des DBC), ainsi que leurs connaissances et compétences en tant que prestataires de planification familiale, leurs valeurs et attitudes envers la planification familiale, et leur engagement vis-à-vis du projet AcQual.

Par ailleurs, l'équipe de recherche a mené des entretiens avec 73 infirmiers formés sous AcQual à la gestion des clientes référencées par les DBC vers les structures de santé statiques, ainsi que les Médecins Chefs de Zone (MCZ) et les Animateurs Communautaires (AC) des bureaux centraux des 33 zones de santé où les DBC d'AcQual étaient actifs.

Toutes les enquêtes quantitatives ont été administrées électroniquement en utilisant l'application sur Smartphone Open Data Kit (ODK), et envoyées sur un serveur sécurisé où les données ont été agrégées et extraites par l'équipe de recherche basée aux États-Unis. Ce processus a permis une restitution extrêmement rapide des résultats de l'évaluation: la collecte de données s'est déroulée en avril 2017, et les résultats ont été présentés lors d'un événement de dissémination en juillet 2017.

Les données de l'enquête des DBC et des infirmiers ont été triangulées avec une analyse systématique des rapports mensuels disponibles aux BCZS pour la période de janvier à mars 2017. Les membres de l'équipe de recherche se sont

rendus dans chaque BCZS et ont relevé le nombre de rapports mensuels de DBC disponibles en comparaison au nombre attendu (chaque DBC devait envoyer deux rapports tous les mois, soit un rapport sur les services fournis et un autre sur les stocks et le flux des contraceptifs).

Enfin, l'équipe de recherche a mené 10 entretiens approfondis avec les partenaires nationaux (le personnel du Ministère de la Santé et les représentants d'ABEF et SANRU) et internationaux (le FNUAP et DKT International, qui étaient chargés de l'approvisionnement du projet en contraceptifs).

La collecte des données pour cette évaluation de processus a été autorisée par le Comité d'éthique biomédicale de l'Université de Tulane (1029921-OTH) ainsi que le Comité d'éthique de l'École de Santé Publique de Kinshasa (#ESP/CE/014/2017).

RÉSULTATS

Sur les 870 DBC formés par les partenaires d'AcQual depuis février 2014, 105 (12,1%) n'étaient plus actifs (dont 88 injoignables et 7 décédés), 65 (7,5%) étaient toujours actifs mais ont refusé d'être interrogés, et 700 (80,5%) ont déclaré être toujours actifs et consenti à être interviewés.

Globalement, le nombre de jours travaillés et d'APC fournies par DBC actifs, d'après leur mémoire du mois écoulé, est faible. Les DBC ont déclaré « faire quelque chose en lien avec le projet AcQual » en moyenne 8 jours par mois. Interrogés sur les quantités de chaque contraceptif qu'ils se souvenaient avoir distribués au cours du mois précédant l'enquête, la quantité moyenne d'APC fournies s'élevait à 3,8, avec une médiane de 1,3 (moins d'un collier du cycle par mois).

Les résultats suivants illustrent en détails les différentes dimensions de la mise en œuvre d'AcQual 2 estimées comme étant cruciales pour la distribution de contraceptifs en grandes quantités et l'offre de services de planification familiale de qualité par les DBC au niveau communautaire: des lacunes ont été identifiées autant dans la conception du modèle que dans sa mise en œuvre.

Une couverture adéquate

AcQual a formé et déployé trois DBC par aire de santé dans 303 des 381 (73,5%) aires de santé de la ville. Le projet a également formé 73 infirmiers additionnels dans les structures de santé de référence (2,2 par zone de santé en moyenne). Ainsi, AcQual a atteint son objectif de couvrir au moins 70% de toutes les aires de santé de Kinshasa. Selon les données démographiques fournies par l'Institut National de la Statistique du Congo, le nombre moyen de DBC était de 12,1 pour 100 000 habitants au niveau des zones de santé (avec une médiane de 9,3).

Une préparation inadéquate pour fournir des services de planification familiale de qualité

Tous les DBC ont été recrutés et ont opéré dans leurs propres quartiers, et en accord avec le modèle AcQual, presque tous (99,3%) avaient terminé au moins l'école primaire, 70,7% ayant achevé le lycée ou des études supérieures. Plus de la moitié (57,1%) étaient sans emploi et un tiers (33,8%) étaient déjà impliqués en tant qu'agents de santé communautaire dans d'autres programmes. L'âge moyen des DBC d'AcQual était de 45,9 ans, et 82,4% avaient plus de 35 ans. Concernant les raisons de leur choix de travailler bénévolement pour AcQual, 42,1% des DBC ont exprimé un désir d'« aider la communauté », tandis que 33,8% ont indiqué être déjà impliqués dans des activités de sensibilisation en matière de santé, et 22,9% ont déclaré vouloir acquérir de nouvelles compétences. Moins de 1% ont mentionné l'argent comme source initiale de motivation.

Les DBC ont tous témoigné à l'unanimité de leur satisfaction quant à la formation dont ils ont bénéficié. Cependant, les résultats du test de connaissances sur 15 questions de planification familiale étaient médiocres, avec un tiers des DBC (32,0%) enregistrant un score en-dessous de la moyenne, et entre un quart et un tiers restituant des informations inexactes sur le diagnostique des grossesses, l'utilisation du collier du cycle et les critères d'éligibilité médicale à la pilule. En plus de ces faibles niveaux de connaissances, les valeurs personnelles (approximées par le degré de confort avec lequel les DBC étaient prêts à distribuer les contraceptifs à certaines populations) pourraient avoir freiné leur niveau d'activité: bien que 96,1% et 84,4% des DBC aient déclaré être « assez ou très à l'aise » en fournissant des contraceptifs aux hommes et aux femmes non mariées, respectivement, un quart d'entre eux (23,4%) se sentaient « assez ou très mal à l'aise » de les vendre à des jeunes de moins de 18 ans, et près de la moitié (47,4%) ont déclaré se sentir « assez ou très mal à l'aise » de fournir des services de planification familiale à des jeunes de moins de 15 ans. (Étant donné que cette question a un fort biais de désirabilité, il se peut que les chiffres réels soient plus élevés encore). Au total, 78,3% des

TABLEAU 1. Contraceptifs en rupture de stock et réapprovisionnés déclarés par les DBC

	Préservatifs	Pilule COC	Pilule POP	Collier du cycle
Souvent en rupture de stock	41,40%	20,3%	22,7%	24,2%
Parfois en rupture de stock	34,0%	30,3%	23,1%	22,6%
Une fois en rupture de stock	10,0%	13,1%	11,3%	13,7%
Jamais en rupture de stock	14,6%	36,2%	42,9%	39,6%
Ruptures de stock déclarées	————————81,30——————————			
Réapprovisionnés	74,9%	70,4%	58,3%	57,2%

DBC auraient aimé suivre une formation de recyclage, particulièrement sur la manière de recruter de nouvelles utilisatrices de la contraception et comment référer les femmes vers des structures de santé pour la gestion des effets secondaires.

Des ruptures de stocks récurrentes

Les ruptures de stocks des contraceptifs constituent l'un des problèmes les plus communément mentionnés par les DBC. Plus de trois quart (77,3%) des DBC avaient fait l'expérience d'une rupture de stock depuis le début du projet, et 75,4% ont déclaré être « parfois » ou « souvent » en rupture de stock de préservatifs, (48,2% pour les pilules et 46,2% pour le collier du cycle - Tableau 1). De plus, seuls 46,7% des DBC ont déclaré avoir au moins un type de documentation pour le conseil en planification familiale (ex: dépliants, aide-mémoires imprimés, etc.) le jour de l'enquête.

Les ruptures de stocks relativement plus rares de la pilule et du collier du cycle pourraient indiquer une demande plus faible de ces types de contraceptifs, une hypothèse soutenue par les préférences des femmes perçues par les DBC dans leurs communautés. Selon les DBC, moins d'un quart de toutes les femmes préfèrent utiliser le Collier du Cycle (23,6%), la pilule (21,7%) ou le préservatif (18,9%) comme première méthode de contraception. Les perceptions des DBC sont cohérentes avec les résultats des enquêtes récentes auprès de la population, qui indiquent de plus hauts niveaux d'utilisation de l'implant, l'injectable et la contraception d'urgence.[2] Les données qualitatives émergeant des entretiens avec les DBC mettent en évidence leur frustration quant à leur incapacité d'offrir ces méthodes, qui ne peuvent traditionnellement être distribuées en RDC que par du personnel de santé ayant suivi une formation médicale.[7]

Une faible interaction avec les services cliniques

L'écart observé par les DBC entre les méthodes qu'ils étaient autorisés à fournir et celles demandées par les utilisatrices potentielles aurait pu être contrebalancé en apportant des modifications au modèle AcQual, pour autant que le système de référence vers les structures de santé AcQual ait été opérationnel et efficace. La majorité des DBC (89,8%) ont déclaré avoir référé « quelques » ou « plusieurs » femmes vers une structure de santé depuis le début du projet, dont 71% vers les structures désignées par les partenaires d'AcQual pendant la formation. Dans la majorité des cas, les femmes étaient référées car la méthode qu'elles désiraient n'était pas fournie par le DBC (Tableau 2). La deuxième raison la plus citée, par 29,9% des DBC, était la gestion des effets secondaires.

Seule la moitié des infirmiers (54,7%) ont pu identifier « tous » ou « la plupart » des DBC opérant dans leurs zones de santé. Mais un plus haut pourcentage (62,8%) a déclaré interagir entre une fois par semaine et une fois par mois avec certains DBC dans leurs zones de santé, ce qui pourrait indiquer que les infirmiers tendent à interagir toujours avec les mêmes personnes, tandis que d'autres DBC ne sont pas surveillés. Globalement, le travail des DBC est perçu comme étant mutuellement bénéfique (par 96,6% des DBC et 87,1% des infirmiers). Parmi les 7,1% d'infirmiers de référence ayant exprimé une opinion « très négative » concernant le travail des DBC, la plupart étaient préoccupés par les erreurs médicales commises par les DBC en administrant les méthodes contraceptives, et les attitudes trompeuses des DBC qui, selon les mots de l'un des infirmiers, « se présentent comme des 'médecins' alors qu'ils n'ont suivi aucune formation adéquate. »

Un faible accompagnement des distributeurs à base communautaire

Pour qu'AcQual atteigne ses objectifs, le projet doit suivre et superviser les activités des DBC

TABLEAU 2. Réponses comparées des DBC et des infirmiers formés par AcQual pour l'évaluation du système de référence

	Entretiens DBC	Entretiens infirmiers
Capable d'identifier son homologue dans le système de référence	88,4%	54,7%
**Interactions entre les DBC et les structures de référence**		
Interagit souvent avec son homologue	13,7%	31,4%
Interagit parfois avec son homologue	34,3%	31,4%
Interagit rarement avec son homologue	42,6%	8,6%
N'interagit jamais avec son homologue	7,3%	27,1%
**Raisons de la référence vers la structure de santé**		
Méthode non fournie par le DBC	83,9%	67,1%
Gestion des effets secondaires	29,9%	40,0%
Préférence pour les structures de santé	11,3%	22,9%
Méthode fournie par le DBC mais en rupture de stock	9,1%	17,1%
Retrait d'implant/DIU	—	21,4%
Autre	7,4%	—
Ne sait pas	3,3%	—
**Perception des activités des DBC par le personnel des structures de santé**		
Très positive	96,6%	70,0%
Assez positive		17,1%
Assez négative	2,1%	0,0%
Très négative		7,1%
Ne sait pas	1,2%	5,7%

dans le but de leur fournir un soutien technique adéquat, d'assurer la rétention et motivation des DBC, de limiter les ruptures de stocks en contraceptifs, et de garantir l'exactitude et la ponctualité des rapports des statistiques de services de routine. Ce dernier point constitue une autre faiblesse de la mise en œuvre d'AcQual: seulement 32,5% des rapports mensuels étaient disponibles au BCZS pour la période de janvier-mars 2017. Seuls 48,4% des DBC savaient qu'ils étaient tenus de remplir deux formulaires par mois (un pour les services de planification familiale, et un autre pour les stocks de contraceptifs, en suivant les formats et conditions du Système d'Information Sanitaire National de la RDC), et 46,9% ont indiqué avoir eu des difficultés à remplir et transmettre leurs rapports au BCZS chaque mois. Les raisons principales mentionnées étaient l'absence de clients (21,2%) et le manque de temps pour remplir (14,4%) ou déposer (15,4%) le formulaire au BCZS. Avec 93,8% des DBC remettant leurs données en version papier, l'éloignement et les frais de transport, particulièrement dans les zones de santé semi-rurales recouvrant une grande superficie dans la périphérie de Kinshasa, ont constitué des barrières importantes à la transmission des statistiques de routine.

Inversement, 55,4% des DBC ont déclaré ne plus avoir de formulaire vierge pour les prochains rapports. Le personnel des BCZS interrogé pendant l'évaluation a suggéré que certaines de ces statistiques pourraient être intentionnellement sous-déclarées, étant donné que les DBC sont supposés partager les recettes de leurs ventes de contraceptifs avec le BCZS et les partenaires d'AcQual. Cette situation pourrait expliquer pourquoi 34,9% du personnel des BCZS a indiqué que les DBC ne déclaraient que « rarement » ou « jamais » leurs ventes de contraceptifs.

Par ailleurs, bien que les DBC aient déclaré être globalement « satisfaits » ou « très satisfaits » (27,8% et 63,2%, respectivement) de leurs relations avec le BCZD, le niveau d'interaction rapportée entre eux semble varier significativement d'une zone de santé à l'autre. La grande majorité (82,3%) des DBC a déclaré avoir assisté à au moins

une réunion de suivi dans le BCZS, mais légèrement plus de la moitié (56,9%) du personnel du BCZS ne savait pas combien de ces réunions avaient été tenues dans les six derniers mois. De plus, un membre du personnel du BCZS sur six a indiqué qu'aucune réunion de suivi n'avait été organisée, et seuls 4,6% ont déclaré que toutes les réunions mensuelles avaient eu lieu comme prévu et en accord avec les directives d'AcQual. À l'échelon de supervision supérieur (les partenaires de mise en œuvre d'AcQual, ABEF et SANRU), seul un tiers (36,6%) des DBC se rappelaient avoir interagi avec le personnel de ces organisations après la formation. Les chiffres sont plus élevés pour le personnel du BCZS (83,3%), à qui ABEF/SANRU devaient normalement rendre visite au moins une fois par mois pour récupérer les rapports des statistiques de services et gérer le réapprovisionnement en contraceptifs. Cependant, interrogé sur le soutien reçu par les partenaires d'AcQual, 12,1% du personnel des BCZS a déclaré qu'il n'était « pas très bon » et 21,2% « pas bon du tout ».

La perception générale du projet par ses partenaires de mise en œuvre principaux demeure positive, avec 78,4% du personnel des BCZS et 97,2% des DBC déclarant être « satisfaits » ou « très satisfaits » de leur expérience d'AcQual. Toutefois, des commentaires qualitatifs à la fin des deux enquêtes ont enregistré des plaintes récurrentes sur le manque de soutien et de motivations adéquates. De nombreux DBC se sont plaint du fait que:

> *On [nous] demande de faire un travail très difficile presque gratuitement [. . .] Nous n'avons pas l'équipement, comme des bottes de pluies ou un parapluie, on ne nous donne pas beaucoup de contraceptifs, et nous n'avons pas de salaire ou assez de reconnaissance pour nous motiver. [. . .] Nous avons besoin de voir de nouvelles personnes s'impliquer. Cela fait presque trois ans, et parfois on a l'impression qu'AcQual nous a un peu abandonnés.*

DISCUSSION ET CHANGEMENTS SUBSÉQUENTS APPORTÉS AU MODÈLE ACQUAL

Un article récent publié dans le *Lancet,* lançant un appel mondial à la transparence dans l'évaluation des projets de santé maternelle, a souligné « l'incongruité entre les succès –invariablement rapportés au niveau discret des programmes– et le manque de progrès collectif dans la lutte contre la mortalité maternelle à l'échelle internationale », et suggéré que la sous-représentation des « résultats malvenus » dans les évaluations de projets pourrait en partie être responsable de cette incohérence.[11] Les résultats présentés dans cet article soulignent les échecs malvenus à la fois de conception et de mise en œuvre qui ont miné la capacité d'AcQual à augmenter l'accès aux services de planification familiale de qualité au niveau communautaire.

Les résultats pointent spécifiquement vers deux types de problèmes: (1) des failles propres au modèle AcQual ou à sa mise en œuvre, qui n'ont pas été anticipées ou contrôlées au lancement du projet, et (2) des problèmes systémiques liés au système de la santé de la RDC, qui nécessiteraient une approche plus ample et multisectorielle au-delà de la portée d'AcQual sur laquelle s'appuyer pour s'améliorer.

Étant donnée l'abondance de la littérature existante sur les agents de santé communautaire,[12–15] certains résultats de cette évaluation ne sont pas particulièrement surprenants (ex: le manque d'engagement à long terme des bénévoles, l'absence de rémunération et la faible intégration des DBC aux structures de santé). Cependant, AcQual est le premier programme de distribution à base communautaire à avoir été mis en œuvre à Kinshasa après un hiatus de 20 ans, et sa conception a été menée en étroite collaboration avec le Ministère de la Santé dans le but de l'intégrer *in fine* au système national de services de planification familiale. Certaines caractéristiques du projet ont donc été définies pour éviter de peser davantage sur un système de santé déjà contraint par des ressources limitées, mais sans être toujours en mesure de compenser les lacunes existantes.

Essentiellement, le postulat initial selon lequel le pourcentage des recettes des ventes de contraceptifs revenant aux DBC constituerait une motivation suffisante pour compenser leur statut de « bénévole », et pour encourager leur proactivité dans la prestation des services de planification familiale dans leurs communautés, s'est vu significativement ébranlé par le prix intentionnellement bas des contraceptifs, accentuant l'absence de motivation à documenter les statistiques de services. Dans de nombreux cas, les bénéfices perçus par les ventes mensuelles des contraceptifs ne permettaient pas de couvrir le coÃt du transport des DBC pour assister aux réunions de suivi au bureau central de la zone de santé. Des intérêts concurrents ont également compliqué cette situation, étant donné que les DBC travaillent aussi souvent en tant qu'agents de santé commentaire pour d'autres projets (ex: immunisation ou prévention du VIH/SIDA) qui les rémunèrent directement.

De plus, les normes médicales existantes en RDC limitent *de facto* la gamme de méthodes offertes par les DBC d'AcQual, lesquelles ne correspondent pas aux préférences des femmes pour les méthodes réversibles à moyenne et longue durée d'action tels que les injectables et les implants. Les DBC et les partenaires d'AcQual ont perçu cette situation comme une opportunité perdue, puisque le prix de vente plus élevé de ces contraceptifs aurait pu soutenir un engagement plus solide des DBC dans l'offre des services communautaires.

Enfin, le modèle de distribution de routine encourageant les DBC à fournir des contraceptifs « dès qu'ils en avaient l'occasion, dans leurs vies quotidiennes, dans leurs communautés » s'est avéré arbitraire en pratique, au vu des niveaux d'activités inégaux des DBC, des failles de la chaîne de réapprovisionnement des contraceptifs et du rapportage. Sur ce point, l'évaluation de processus a également fourni des preuves permettant à toutes les parties prenantes d'AcQual de reconnaître et discuter des changements organisationnels nécessaires, dont l'identification et le remplacement des DBC improductifs, la formation de recyclage des DBC actifs (y compris une clarification des valeurs sur la prestation des services de planification familiale à des segments de la population tels que les jeunes et les femmes non mariées), la formalisation des responsabilités de supervision du BCZS et des chaînes de rapportage jusqu'à l'équipe de direction d'AcQual à Tulane International, ainsi que la réallocation de rubriques budgétaires pour augmenter la couverture des activités de suivi et supervision.

Changements apportés au modèle dans le cadre d'AcQual 3

De manière plus cruciale, les résultats de l'évaluation de processus ont informé les changements de conception et de mise en œuvre qui ont mené à la révision du modèle de distribution à base communautaire devant être mis en œuvre à Kinshasa et dans la province du Kongo Central en 2019–2021 (AcQual 3). En particulier, les DBC sont désormais organisés selon un emploi du temps de prestation de services plus rigoureux, rythmé par des événements de mini-campagnes mensuelles appelés « Samedi PF ». Cette approche fait d'AcQual 3 une stratégie proche de celles des journées de campagne/stratégies avancées mises en œuvre par les partenaires nationaux et internationaux en RDC comme principale méthode de distribution à base communautaire. Les Samedis PF devraient créer un événement catalyseur pour impliquer les DBC en réunissant plusieurs prestataires à base communautaire en un seul lieu (généralement, une place de marché), et donc attirer potentiellement plus de clients. De plus, ces événements augmenteront la visibilité des DBC dans la communauté en les présentant comme une ressource locale pour le conseil et l'offre de méthodes de planification familiale, tout en leur donnant l'opportunité de rapporter leurs statistiques de services, de se réapprovisionner en contraceptifs, et d'être supervisés sans frais de transport additionnels.

De plus, les recettes des ventes de contraceptifs seront désormais entièrement versées aux DBC pour renforcer leur motivation financière à participer aux activités d'AcQual. Ce changement devrait être couplé d'une expansion de la gamme de méthodes offertes par les DBC, qui sous AcQual 3 incluent le DMPA-SC (Sayana Press) et la contraception d'urgence, tous deux en forte demande et à un prix de vente plus élevé que les autres méthodes fournies via la distribution à base communautaire. L'ajout de ces deux méthodes à la liste des contraceptifs fournis par les DBC est le résultat d'une recherche séparée menée par Tulane, qui a mis en évidence les opportunités de distribution à base communautaire de ces méthodes,[16,17] et illustré comment les études pilotes et la recherche opérationnelle (telle que cette évaluation de processus) peuvent être utilisées pour améliorer la conception et la mise en œuvre des programmes.[18]

Cependant, certains des défis identifiés dans l'évaluation de mi-parcours dépassent en partie la portée du seul projet AcQual. Les pénuries chroniques et les failles enregistrées dans la chaîne d'approvisionnement des contraceptifs demeurent un problème récurrent en RDC, d'autant plus dans un contexte où la demande contraceptive est en augmentation depuis plusieurs années. Les structures de santé et les agents de santé communautaire tendent à être surchargés par de nombreuses responsabilités et exigences de rapportage sur les programmes et projets.[19] En dépit des efforts entrepris pour intégrer les activités d'AcQual aux programmes de santé mis en œuvre au niveau des zones de santé (ex: se reposer sur le BCZS pour le recrutement des DBC, impliquer le Ministère de la Santé pour leur formation, utiliser les formulaires standards pour le rapport de statistiques), les activités du projet, les protocoles de réapprovisionnement en contraceptifs, et les besoins de rapport ont généré des redondances et des exigences concurrentes faisant pression sur

l'énergie et le temps déjà limités du personnel de santé. Les prochaines modifications de l'approche AcQual devraient chercher à « augmenter l'appropriation et le leadership du gouvernement, limiter les apports externes, et institutionnaliser les interventions au sein des structures existantes. »[20]

Limites

La littérature existante sur les évaluations de processus s'accorde à souligner l'absence de cadre d'analyse systématique[9,10] et le volume d'information écrasant, et possiblement redondant, qu'elles tendent à générer. Cette étude a tenté de comparer la performance réelle du programme à son modèle de fonctionnement escompté. Pour ce faire, l'équipe de recherche a mesuré la fidélité au modèle tel qu'il a été conçu, et la préparation des prestataires à fournir ces services, leurs perceptions et leur degré de satisfaction. En revanche, cette étude n'explore pas d'autres dimensions qui peuvent faire l'objet des évaluations de processus, comme l'exposition et la satisfaction des clients.

Par ailleurs, le manque de données sur la performance individuelle de chaque DBC (mesurée par le volume de contraceptifs distribués) a contraint l'équipe de recherche à se reposer sur des indicateurs quantitatifs ad hoc créés pour cette évaluation (nombre de jours travaillés, volume de méthodes fournies, et score de connaissances) fondés sur des données fournies par les DBC eux-mêmes à partir de leur remémoration, ce pourquoi ces données pourraient être biaisées en tendant soit vers une surreprésentation de leurs activités de distribution de contraceptifs (biais de désirabilité), soit vers leur sous-représentation (si les DBC ont pensé que ceci attirerait plus de soutien direct, ou s'ils n'ont pas déclaré leurs ventes pour en garder les bénéfices). Les lacunes enregistrées dans le rapportage et la supervision de la prestation de services de planification familiale pourraient en elles-mêmes contribuer à la faible performance perçue des DBC.

De plus, l'accent mis sur les APC comme mesure du succès de la mise en œuvre d'AcQual néglige certaines dimensions de la qualité des services (filtrage des clients pour la planification familiale et conseil adéquat), lesquelles sont cruciales non seulement pour l'adoption de méthodes de contraception, mais aussi pour leur utilisation sur le long terme.

Enfin, cette focalisation sur les APC pourrait aussi négliger d'autres aspects importants du travail des DBC dans la promotion de l'accès aux contraceptifs (ex: sensibilisation, conseil individualisé) et pour augmenter autant les connaissances que la demande de services de planification familiale dans la communauté.

CONCLUSION

L'environnement de l'offre de planification familiale à Kinshasa évolue rapidement depuis 2012.[21–23] Les efforts de nombreux partenaires nationaux et internationaux dans ce domaine se recoupent, et sont parfois en concurrence, en utilisant différentes stratégies de tarification, échelles d'intervention, et sites de services communautaires ou cliniques. Il en résulte une impossibilité d'attribuer la lente, mais néanmoins constante, augmentation du TPCm à Kinshasa à un projet ou programme en particulier, et AcQual ne fait pas exception. Toutefois, la préférence donnée à une évaluation de processus du projet, et la présentation de ses résultats à l'ensemble des partenaires de la planification familiale opérant à Kinshasa en juillet 2017, a permis d'isoler et d'évaluer des composantes de l'intervention communes à d'autres projets ou programmes (ex: utilisation de différents cadres d'agent de santé communautaire, approche de marketing social de la tarification des contraceptifs) et de clarifier leurs interactions, tout en identifiant les facteurs environnementaux et les réponses associées à la variation des résultats du projet.

Dans le cas d'AcQual 2, en plus des failles systémiques du système de santé de la RDC, des échecs à la fois de conception (gamme de méthodes inappropriée et modèle de motivation inadéquat pour maintenir l'engagement des DBC) et de mise en œuvre (failles de la logistique contraceptive et insuffisance du suivi et supervision des activités des DBC) sont responsables de la faible performance du projet en termes d'APC fournies. Une fois ces failles identifiées, Tulane et ses partenaires ont utilisé les résultats de l'évaluation pour informer les modifications clés apportées à la conception et l'application du modèle, aujourd'hui AcQual 3, actuellement mis en œuvre en RDC (2019–2021).

Dans un contexte où le passage à échelle et la durabilité des modèles de distribution à base communautaire de la planification familiale dans des environnements aux ressources limitées soulèvent de plus en plus de questions, des évaluations de processus comme celle ici présentée constituent d'importantes contributions. Elles démontrent comment les données scientifiques, les stratégies des bailleurs de fonds, et les situations locales et nationales informent les choix de conception initiaux, qui après avoir été testés sur le terrain

peuvent s'avérer inadaptés. L'innovation et les leçons partagées par d'autres projets peuvent aider à corriger certaines de ces lacunes identifiées. Toutefois, l'intégration réaliste de ces modèles aux systèmes de santé nationaux (au-delà du succès de projets pilotes autonomes et bénéficiant de ressources conséquentes) nécessitera de trouver des compromis inventifs entre le renforcement des éléments les plus efficaces du modèle et l'adaptation aux défaillances systémiques chroniques.

Remerciements: Les auteurs souhaitent exprimer leur gratitude envers la Fondation David et Lucie Packard pour leur soutien au travail décrit dans cet article. En particulier, nous remercions Tamara Kreinin, Coley Gray, Kristina Kastler et Amy Iftekhar.

Financement: Fondation David et Lucile Packard (Subvention #2017/65766).

Conflit d'intérêt: Rien à déclarer.

BIBLIOGRAPHIE

1. Ministère du Plan et Suivi de la Mise en œuvre de la Révolution de la Modernité (MPSMRM); Ministère de la Santé Publique (MSP); ICF International. *Enquête Démographique et de Santé en République Démocratique du Congo 2013-2014.* Rockville, MD: MPSMRM, MSP, and ICF International; 2014. https://dhsprogram.com/publications/publication-FR300-DHS-Final-Reports.cfm. Accessed December 4, 2018.
2. Performance Monitoring and Accountability (PMA2020). *PMA2016/Kinshasa-R5.* Baltimore, MD: PMA2020; 2016. https://pma2020.org/sites/default/files/DRCR5-KINSHASA-EN-FP-Brief-v6-2017.01.23.pdf. Accessed December 4, 2018.
3. Herp MV, Parqué V, Rackley E, Ford N. Mortality, violence and lack of access to healthcare in the Democratic Republic of Congo. *Disasters.* 2003;27(2):141–153. CrossRef. Medline
4. Feinstein L, Dimomfu BL, Mupenda B, et al. Antenatal and delivery services in Kinshasa, Democratic Republic of Congo: care-seeking and experiences reported by women in a household-based survey. *Trop Med Int Health.* 2013;18(10):1211–1221. CrossRef. Medline
5. Phillips JF, Greene WL, Jackson EF. Lessons from community-based distribution of family planning in Africa. New York: Population Council; 1999. http://chwcentral.org/sites/default/files/Lessons%20from%20Community-based%20Distribution%20of%20Family%20Planning%20in%20Africa.pdf. Accessed December 4, 2018.
6. Bertrand JT, McBride ME, Mangani N, Baughman NC, Kinuani M. Community-based distribution of contraceptives in Zaire. *Int Fam Plan Perspect.* 1993;19(3):84–91. CrossRef
7. Medhanyie A, Spigt M, Kifle Y, et al. The role of health extension workers in improving utilization of maternal health services in rural areas in Ethiopia: a cross sectional study. *BMC Health Serv Res.* 2012;12(1):352. CrossRef. Medline
8. Bertrand JT, Makani PB, Hernandez J, et al. Acceptability of the community-level provision of Sayana® Press by medical and nursing students in Kinshasa, Democratic Republic of the Congo. *Contraception.* 2017;96(3):211–215. CrossRef. Medline
9. Moore GF, Audrey S, Barker M, et al. *Process evaluation of complex interventions: Medical Research Council guidance. BMJ.* 2015;350:h1258. CrossRef. Medline
10. Steckler AB, Linnan L, Israel BA. *Process Evaluation for Public Health Interventions and Research.* San Francisco, CA: Jossey-Bass; 2002.
11. Mumtaz Z, Ellison GTH, Ferguson A, Salway S. A call for transparency in the evaluation of global maternal health projects. *Lancet.* 2016;388(10043):461–461. CrossRef. Medline
12. Rahman SM, Ali NA, Jennings L, et al. Factors affecting recruitment and retention of community health workers in a newborn care intervention in Bangladesh. *Hum Res Health.* 2010;8:12. CrossRef. Medline
13. Dolea C, Stormont L, Braichet JM. Evaluated strategies to increase attraction and retention of health workers in remote and rural areas. *Bull World Health Organ.* 2010;88(5):379–385. CrossRef. Medline
14. Teklehaimanot A, Kitaw Y, Yohannes AG, et al. Study of the working conditions of health extension workers in Ethiopia. *Ethiop J Health Dev.* 2007;21(3):246–259. CrossRef
15. Lehmann U, Friedman I, Sanders D. Review of the utilization and effectiveness of community-based health workers in Africa. https://pdfs.semanticscholar.org/7a0c/fd44975b00b045c7957c9327a87c9fcc6b63.pdf. Published 2004. Accessed June 1, 2018.
16. Mwembo A, Emel R, Koba T, et al. Acceptability of the distribution of DMPA-SC by community health workers among acceptors in the rural province of Lualaba in the Democratic Republic of the Congo: a pilot study. *Contraception.* 2018;98(5):454–459. CrossRef. Medline
17. Hernandez JH, Muanda M, Garcia M, Matawa G. Awareness and perceptions of emergency contraceptive pills among women in Kinshasa, Democratic Republic of the Congo. *Int Perspect Sex Reprod Health.* 2017;43(3):121–130. CrossRef. Medline
18. Binanga A, Bertrand JT. Pilot research as advocacy: the case of Sayana Press in Kinshasa, Democratic Republic of the Congo. *Glob Health Sci Pract.* 2016;4(4):542–551. CrossRef. Medline
19. Bertone MP, Lurton G, Mutombo PB. Investigating the remuneration of health workers in the DR Congo: implications for the health workforce and the health system in a fragile setting. *Health Policy Plan.* 2016;31(9):1143–1151. CrossRef. Medline
20. Keyonzo N, Nyachae P, Kagwe P, et al. From project to program: Tupange's experience with scaling up family planning interventions in urban Kenya. *Reprod Health Matters.* 2015;23(45):103–113. CrossRef. Medline
21. Babazadeh S, Lea S, Kayembe P, et al. Assessing the contraceptive supply environment in Kinshasa, DRC: trend data from PMA2020. *Health Policy Plan.* 2018;33(2):155–162. CrossRef. Medline
22. Mukaba T, Binanga A, Fohl S, Bertrand JT. Family planning policy environment in the Democratic Republic of the Congo: levers of positive change and prospects for sustainability. *Glob Health Sci Pract.* 2015;3(2):163–173. CrossRef. Medline
23. Kwete D, Binanga A, Mukaba T, et al. Family planning in the Democratic Republic of the Congo: encouraging momentum, formidable challenges. *Glob Health Sci Pract.* 2018;6(1):40–54. CrossRef. Medline

Revu par les pairs

Reçu : juin 1, 2018; **Accepté :** novembre 24, 2018

© Hernandez et al. This is an open-access article distributed under the terms of the Creative Commons Attribution License, which permits unrestricted use, distribution, and reproduction in any medium, provided the original author and source are properly cited. To view a copy of the license, visit http://creativecommons.org/licenses/by/4.0/. When linking to this article, please use the following link: https://doi.org/10.9745/GHSP-D-18-00205
